# Doctors of osteopathic medicine (DO): a Canadian perspective

**Published:** 2014-12-17

**Authors:** Sevan Evren, Andrew Yuzhong Bi, Shuchi Talwar, Andrew Yeh, Howard Teitelbaum

**Affiliations:** 1 Dept. of Preventive Medicine, Lincoln Memorial University, Tennessee, United States; 2 Dept. of Obstetrics and Gynecology, Western University of Health Sciences, California, United States

## Abstract

**Background:**

Doctors of osteopathic medicine (DO) are one of the fastest growing segments of health care professionals in the United States. Although Canada has taken significant leaps in the acknowledgment of US trained DOs, there continues to be a lack of understanding of the profession by Canadian trained physicians. In this article, we provide a brief overview of osteopathic medical education and training in the United States.

**Method:**

Current information of osteopathic training by American Association of Colleges of Osteopathic Medicine (AACOM) and American Osteopathic Association (AOA) was presented. Data pertaining to Canadians enrolled in osteopathic colleges was compared with allopathic (MD) and international medical graduates (IMGs).

**Results:**

Doctors of osteopathic medicine programs provide an additional pathway for students interested in pursuing a medical education. Canadian applications to osteopathic colleges are expected to grow due to successful post-graduate US residency matching, increased difficulty of matriculating at Canadian medical schools, and a greater awareness of the profession in Canada.

**Conclusions:**

Given the increasing enrollment of Canadian students in US osteopathic medical schools, we expect that Canadian DOs will play a significant role in shaping health care in both the US and Canada.

## Introduction

Since September 2012, the provinces of Quebec and Manitoba have joined Ontario, British Columbia, and Alberta in recognizing the right of Canadian graduates holding a United States (US) Doctor of Osteopathic Medicine (DO) to participate in the Canadian Resident Matching Service (CaRMS).^1^ The Canadian medical education system has taken significant leaps in the acknowledgment of US trained DOs. However, in spite of this recent progress, Canadian trained physicians remain unaware of osteopathic medical doctors in the clinical setting. In this article we provide a brief overview of osteopathic medical education and training in the United States. In addition, due to an expected increase in the enrollment of Canadian students in osteopathic medical colleges and an increase in collaboration between CMGs and the growing number of practicing DOs and osteopathic affiliated hospitals, we highlight why it is important for Canadian medical doctors to gain an awareness of the osteopathic medical profession.

In the United States there are two types of complete physicians—DOs (Doctor of Osteopathic Medicine) and MDs (Doctor of Allopathic Medicine). Both physicians are trained in the full spectrum of clinical and medical sciences and are allowed to practice in any specialty, prescribe any medication, and perform surgery.^2^ More importantly, DOs and MDs share equal rights.^2^–^4^ In addition to the basic medical science curriculum present in both MD and DO schools, DOs complete an additional +100 hours of training in osteopathic manipulative therapy (OMT) - a hands-on approach to treat the body’s musculoskeletal system.^2^–^4^ Osteopathic physicians train with a greater holistic approach and tactile appreciation of the anatomy of the human body relative to their allopathic counterparts. Osteopathic physicians have traditionally served as primary care physicians in the United States. Osteopathic students are required to take and pass a national board examination, known as the Comprehensive Osteopathic Medicine Licensing Examination (COMLEX). Allopathic graduates are required to take and pass the United States Medical Licensing Examination (USMLE). It is not uncommon for osteopathic students to take both the COMLEX and the USMLE when applying for post graduate training.

Upon successful completion of medical school and post-graduate training, DOs are granted full medical practice rights in the US and in all provinces of Canada, except for Saskatchewan and Prince Edward Island. Osteopathic physicians have the additional benefit of entering post-graduate training programs with either the American Osteopathic Association (AOA) or the Accreditation Council for Graduate Medical Education (ACGME). To date, there are currently 70,000 MDs in Canada. In the US alone, there are over 63,000 active osteopathic physicians in practice.^5^ There are currently 30 accredited US colleges of medicine, spread over 42 teaching sites that matriculate over 5,300 first-year medical students. By 2015 it is expected that 1 in every 4 graduating physicians in the US will be a DO.^4^,^5^

According to the American Association of Colleges of Osteopathic Medicine (AACOM),^6^ the number of Canadian students applying and matriculating into Osteopathic medical colleges has seen a sharp increase (Table 1). In fact, 2014 saw the largest number of Canadian applicants for osteopathic medical schools at 210.^6^ These numbers are expected to grow as we continue to experience an increase in provincial recognition and awareness of osteopathic trained physicians.

Enrollment into Canadian medical schools has become increasingly difficult and has forced many students to seek medical education abroad.^7^ However, the increase in international medical enrollment has exceeded the number of post graduate training opportunities in Canada.^8^–^10^ Consequently, many Canadian graduates of international schools are forced to secure residency programs in the United States but have had very limited success;^7^–^9^ of the 5,133 non-US citizen international medical graduates (IMGs) who applied for ACGME postgraduate training in 2014, approximately 2,411 (47.0%) failed to secure a residency position.^11^ As a result, IMGs including Canadian graduates are burdened with extensive school debt and lack practice privileges. When compared to DOs participating in the match, 2,127 of the 2,738 students (77.7%) were able to secure an ACGME residency position in the US (Table 2).^11^ Additionally, Canadian graduates of American medical schools, DO or MD, are given an additional year of visa sponsorship, known as an optional practical training year (OPT), making the transition toward a residency program easier. Furthermore, 27 out of 41 (66%) of US medical graduates were able to successfully match into a Canadian residency program versus only 449 of the 2318 (19.4%) IMGs.^8^ These data, however, do not differentiate between MDs or DOs returning to Canada. The ability to secure proper ACGME post-graduate training in the United States continues to make osteopathic medical degrees highly attractive to Canadian students versus traditional IMG programs.

## Conclusion

The Doctors of Osteopathic Medicine (DO) programs provide an additional pathway for students interested in pursuing a medical education. The additional benefits associated with a DO degree, including securing proper post-graduate training, will continue to encourage more Canadian students to apply to an osteopathic medical school versus traditional IMG routes. Given the already increasing trend of Canadian enrollment in US osteopathic medical schools and the ongoing advocacy of osteopathic education, it is to be expected that provincial recognition will continue to grow, and that Canadian medical programs will continue to be exposed to osteopathic medical doctors. It is therefore advisable for current medical program directors and doctors in Canada to gain awareness of osteopathic trained physicians.

## Figures and Tables

**Table 1:** The annual number of Canadian applicants to osteopathic medical colleges

Number of Canadian Applicants	2009	2010	2011	2012	2013	2014
Number of Canadian DO Applicants	66	84	114	145	175	210
Number of Canadian DO Matriculates	13	10	40	37	58	pending

**Table 2:** Match statistics for 2014 ACGME post-graduate training in the US

Graduates of Allopathic Medical Schools	Graduates of Osteopathic Medical Schools	Non-US citizen medical School Graduates	Graduates of Canadian Medical Schools
93.7% 16,390/17,487	75.4% 2,019/2,677	47.6% 3,601/7,568	66.7% 14/21
